# Relative Remission and Low Disease Activity Rates of Tofacitinib, Baricitinib, Upadacitinib, and Filgotinib versus Methotrexate in Patients with Disease-Modifying Antirheumatic Drug-Naive Rheumatoid Arthritis

**DOI:** 10.1159/000527186

**Published:** 2023-08-17

**Authors:** Young Ho Lee, Gwan Gyu Song

**Affiliations:** Division of Rheumatology, Department of Internal Medicine, Korea University College of Medicine, Seoul, Republic of Korea

**Keywords:** Janus kinase inhibitors, Low disease activity, Rheumatoid arthritis, Remission, Network meta-analysis

## Abstract

**Background:**

The relative efficacy of Janus kinase (JAK) inhibitors in producing remission and low disease activity (LDA) states remains unknown since there are currently no trials that provide direct comparisons among JAK inhibitors in disease-modifying antirheumatic drug (DMARD)-naive patients with rheumatoid arthritis (RA).

**Objectives:**

This study aimed to assess the relative remission and LDA rates of tofacitinib, baricitinib, upadacitinib, and filgotinib compared to those of methotrexate (MTX) in DMARD-naive patients with RA.

**Method:**

We conducted Bayesian network meta-analysis and included information from direct and indirect comparisons from randomized controlled trials that examined remission (Disease Activity Score in 28 Joints using C-reactive protein level [DAS28-CRP] <2.6) and LDA (DAS28-CRP ≤ 3.2) produced by tofacitinib, baricitinib, upadacitinib, filgotinib monotherapy, and MTX in patients with DMARD-naive RA.

**Results:**

Four randomized controlled trials, comprising 2,185 patients, met the inclusion criteria. This network meta-analysis showed that treatment with tofacitinib, baricitinib, upadacitinib, and filgotinib achieved a significantly higher remission rate than that with MTX (odds ratio [OR] = 4.13, 95% CI = 2.88–6.02; OR = 2.12, 95% CI = 1.17–4.13; OR = 1.95, 95% CI = 1.10–3.50; OR = 1.79, 95% CI = 1.27–3.53). The ranking probability based on the surface under the cumulative ranking curve indicated that upadacitinib 15 mg had the highest probability of achieving remission (SUCRA = 0.985), followed by tofacitinib 5 mg (SUCRA = 0.574), baricitinib 4 mg (SUCRA = 0.506), filgotinib 200 mg (SUCRA = 0.431), and MTX (SUCRA = 0.004). Moreover, treatment with tofacitinib, baricitinib, upadacitinib, and filgotinib achieved significantly higher LDA rate than that with MTX. The ranking probability for LDA was similar to that for remission; upadacitinib 15 mg had the highest probability of achieving LDA, followed by tofacitinib 5 mg, baricitinib 4 mg, filgotinib 200 mg, and MTX.

**Conclusions:**

Upadacitinib seems to be one of most effective interventions for achieving remission and LDA in DMARD-naive patients with RA based on the comparative analysis, and there are differences in remission and LDA rates induced by different JAK inhibitors.

## Introduction

Rheumatoid arthritis (RA) is a systemic autoimmune disease characterized by the persistent inflammation of synovial joints, resulting in disability and poor quality of life [1]. Methotrexate (MTX) is a frequently used disease-modifying antirheumatic drug (DMARD) for treating RA [2]. Nonetheless, not all patients respond to the drug, and 30% of patients discontinue MTX treatment within a year of initiation, mostly due to lack of efficacy or unfavorable side effects [3]. Therefore, therapeutic alternatives to MTX are necessary in patients with RA who are unable to take or tolerate MTX, even though current recommendations support MTX as the first-line DMARD for treating RA [4, 5].

Janus kinases (JAKs), including JAK1, JAK2, JAK3, and tyrosine kinase 2 (Tyk2) are essential for intracellular signaling pathways involved in immune cell activation, pro-inflammatory cytokine synthesis, and cytokine signaling [6]. Tofacitinib, an orally administered JAK inhibitor, has been clinically developed as a small-molecule JAK inhibitor for treating RA [7]. It inhibits JAK1 and JAK3 and, to a lesser extent, JAK2 [8, 9]. Another JAK inhibitor is baricitinib, which is strong and selective against JAK1 and JAK2 [10], but is less effective against JAK3 and Tyk2 [6]. Other JAK inhibitors, upadacitinib and filgotinib, have been designed to be more selective against JAK1 than against JAK2, JAK3, and Tyk2 [11].

Several clinical studies have evaluated the effectiveness of tofacitinib, baricitinib, upadacitinib, and filgotinib (in comparison to that of MTX) in DMARD-naive patients with RA. All these drugs have demonstrated significant efficacy compared to that of MTX; however, their relative efficacy in producing remission and low disease activity (LDA) states remains unknown since there are currently no trials that provide direct comparisons among JAK inhibitors in DMARD-naive patients with RA [12, 13, 14, 15].

Therefore, it is critical to identify the most effective treatment for these individuals. In the absence of head-to-head trials of the relevant comparators, information from randomized controlled trials (RCTs) of various therapies must be combined to assess the relative impact of each therapy [16, 17, 18]. In this investigation, we conducted network meta-analysis to compare the relative remission and LDA rates of tofacitinib, baricitinib, upadacitinib, and filgotinib monotherpy compared to those of MTX in DMARD-naive patients with RA.

## Materials and Methods

### Identification of Eligible Studies and Data Extraction

We performed an exhaustive search for studies that compared the efficacy and safety of treatment with tofacitinib, baricitinib, upadacitinib, or filgotinib monotherpy and MTX in DMARD-naive patients with active RA. A literature search was performed using the MEDLINE, EMBASE, and Cochrane Controlled Trials Register databases and American College of Rheumatology (ACR) and European League against Rheumatism (EULAR) conference proceedings to identify available articles (published until December 2021). The following keywords and subject terms were used for the search: “tofacitinib,” “baricitinib,” “upadacitinib,” “filgotinib,” “DMARD-naive,” and “rheumatoid arthritis.” All references cited in the studies were manually reviewed to identify any additional reports that were not included in the electronic databases. RCTs were included if they met the following criteria: (1) the study compared tofacitinib, baricitinib, upadacitinib, or filgotinib to MTX for the treatment of DMARD-naive patients with active RA; (2) the study provided endpoints for the clinical efficacy of tofacitinib, baricitinib, upadacitinib, or filgotinib at 24 weeks; and (3) the study included patients diagnosed with RA based on the ACR criteria for RA [19] or the 2010 ACR/EULAR classification criteria [20]. The exclusion criteria were as follows: (1) the study included duplicate data and (2) the study did not contain adequate efficacy and safety data for inclusion. The primary endpoint for efficacy was the number of patients who achieved remission (Disease Activity Score in 28 Joints using C-reactive protein level [DAS28-CRP] < 2.6) [21, 22]. The secondary endpoint for efficacy was the number of patients who achieved LDA (DAS28-CRP ≤ 3.2) [23]. Data were extracted from original studies by two independent reviewers. Any discrepancies between them were resolved by consensus. The following information was extracted from each study: first author; year of publication; administered doses of tofacitinib, baricitinib, upadacitinib, filgotinib, or adalimumab; 24-week outcomes; and modified total Sharp score (mTSS). We assessed the methodological quality score for the studies that met the inclusion criteria using the Jadad scale [24]; the quality was classified as high (score of 3–5) or low (score of 0–2). Subsequently, we conducted network meta-analysis following the guidelines provided by the PRISMA statement [25].

### Evaluation of Statistical Associations for Network Meta-Analysis

For multiple-armed RCTs that compared doses of tofacitinib, baricitinib, upadacitinib, and filgotinib in different arms, the results were analyzed simultaneously. The efficacy profiles of tofacitinib, baricitinib, upadacitinib, filgotinib, and MTX in these arms were arranged according to the probability that the treatment would be ranked as the best-performing regimen. We adopted Bayesian fixed-effects model using NetMetaXL [26] and WinBUGS statistical analysis program version 1.4.3 (MRC Biostatistics Unit, Institute of Public Health, Cambridge, UK) for network meta-analysis. We ran the Markov chain Monte Carlo model with 10,000 burn-ins, followed by 10,000 monitoring iterations to obtain pooled-effect sizes [27]. NetMetaXL analysis was conducted by checking whether the Monte Carlo error was less than 5% of the standard deviations of the effect estimates and between-study variance. Information on the relative effects was converted into probability that a treatment was the best, second-best, and so on or into a ranking for each treatment based on the surface under the cumulative ranking curve (SUCRA) [28]. The SUCRA was expressed as a percentage (for example, SUCRA was assigned a value of 100% when a treatment was the best and a value of 0% when a treatment was the worst). League tables were used to organize the summary estimates by ranking the treatments according to the strength of their impact on the outcome based on their SUCRA values [28]. We reported the pairwise comparisons of odds ratios (OR) and 95% credible interval (CrI or Bayesian confidence interval) and adjusted them for multiple-armed trials. The pooled results were considered statistically significant when 95% CrI did not include 1.

### Inconsistency and Sensitivity Tests

Assessment of inconsistency, which refers to the extent of the discord between direct and indirect evidence [29], is important in network meta-analysis [30]. To assess the inconsistency estimates in each loop, we plotted the posterior mean deviance of individual data points in the inconsistency model against their posterior mean deviance in the consistency model [31]. A sensitivity test was performed by comparing the fixed- and random-effects models.

## Results

### Studies Included in the Meta-Analysis

**Fig. 1:**
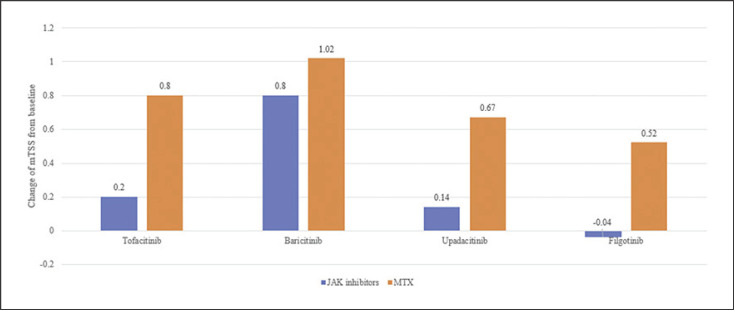
Comparison of mean change in mTSS between JAK inhibitors and MTX.

A total of 257 studies were identified through electronic or manual searches. Among these, 18 studies were selected for full-text review based on the title and abstract details. However, 14 studies being duplicate or irrelevant were further excluded. Finally, four RCTs, including 2,185 patients, met the inclusion criteria [12, 32, 33, 34]. The search results contained 10 pairwise comparisons, including five interventions (Table 1). The Jadad score of the studies was between 3 and 4, indicating high quality. The relevant features of the studies included in the meta-analysis are listed in Table 1. The mean change in mTSS from the baseline to 6-month follow-up was significantly lower in all JAK inhibitors except baricitinib than that in MTX (Fig. 1).

### Network Meta-Analysis of Remission of Tofacitinib, Baricitinib, Upadacitinib, and Filgotinib

**Fig. 2:**
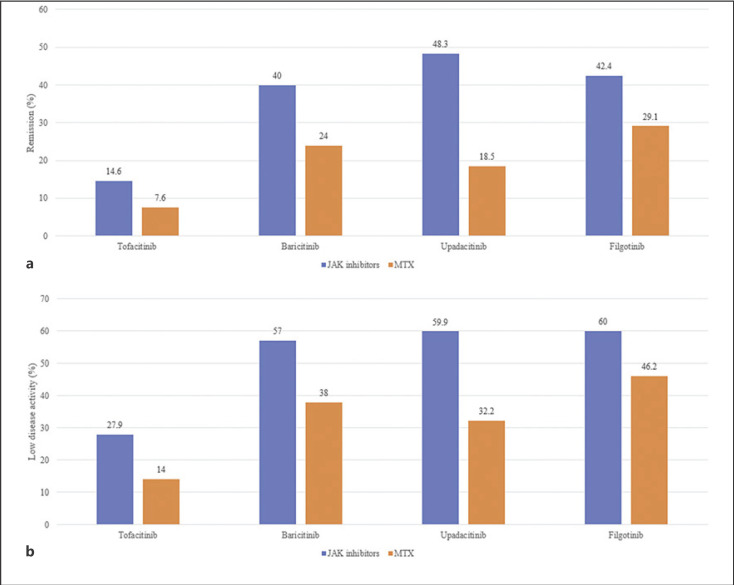
Comparison of remission (**a**) and LDA (**b**) between JAK inhibitors and MTX.

Treatment with tofacitinib, baricitinib, upadacitinib, and filgotinib achieved a significantly higher remission rate than that with MTX (Table 2; Fig. 2). Upadacitinib 15 mg is listed at the top left of the diagonal of the league table (OR, 4.13; 95% CrI, 2.88–6.02) because it was associated with the most favorable SUCRA value of the remission rate, whereas MTX is listed at the bottom right of the diagonal of the league table because it was associated with the least favorable SUCRA value (Tables 2, 3). SUCRA helps in decision-making as the value represents the effect of each treatment as a single number. The ranking probability based on SUCRA indicated that upadacitinib 15 mg had the highest probability of achieving remission, followed by tofacitinib 5 mg, baricitinib 4 mg, filgotinib 200 mg, and MTX (Table 3).

### Network Meta-Analysis of LDA of Tofacitinib, Baricitinib, Upadacitinib, and Filgotinib

**Fig. 3:**
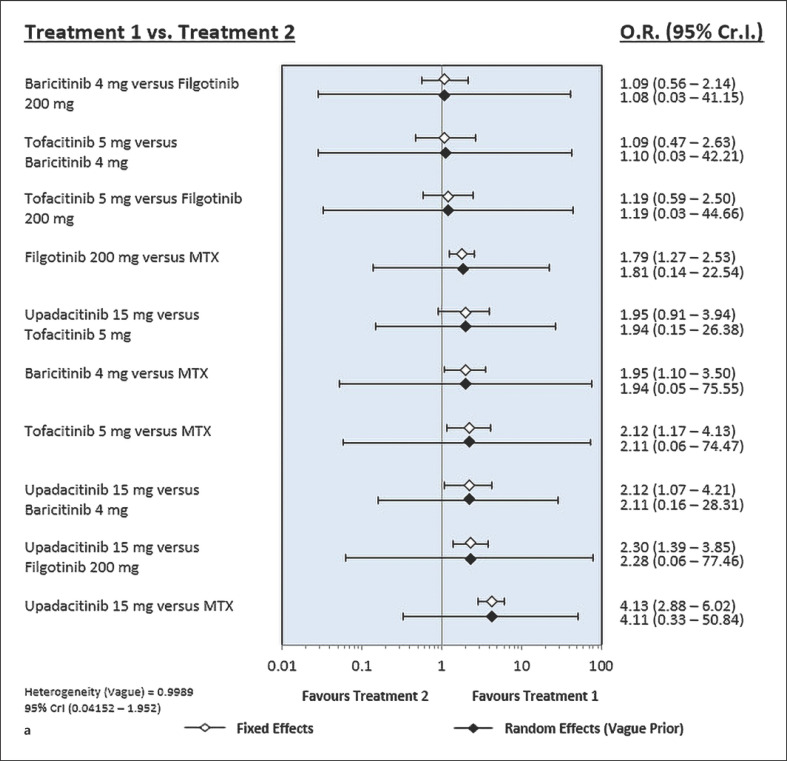
Results of Bayesian network meta-analysis of randomized controlled studies evaluating the relative efficacy of tofacitinib, baricitinib, upadacitinib, filgotinib, or placebo based on the number of patients who achieved remission (**a**) and LDA (**b**).

Treatment with tofacitinib, baricitinib, upadacitinib, and filgotinib achieved a significantly higher LDA rate than that with MTX (Table 2; Fig. 3). Upadacitinib 15 mg is listed at the top left of the diagonal of the league table (OR, 3.17; 95% CrI, 2.29–4.42), whereas MTX is listed at the bottom right of the diagonal of the league table because it was associated with the least favorable SUCRA value (Tables 2, 3). The ranking probability based on SUCRA indicated that upadacitinib 15 mg had the highest probability of achieving LDA, followed by tofacitinib 5 mg, baricitinib 4 mg, filgotinib 200 mg, and MTX (Table 3).

### Inconsistency and Sensitivity Analysis

Inconsistency plots showed a low possibility of inconsistency between the direct and indirect estimates that might significantly affect the results of network meta-analysis. In addition, the random-effects model provided the similar interpretation to that of the fixed-effects model, indicating robustness of the results of network meta-analysis.

## Discussion

We conducted network meta-analysis in DMARD-naive patients with active RA to examine the remission and LDA rates of JAK inhibitors (tofacitinib, baricitinib, upadacitinib, and filgotinib). Regarding remission and LDA rates, the four JAK inhibitors outperformed MTX by substantially increasing the rates of DAS28-CRP < 2.6 and DAS28-CRP ≤ 3.2, and therefore, JAK inhibitor therapy has been suggested to show greater response rates of remission and LDA than those of MTX therapy. Thus, therapy with either of the four JAK inhibitors is successful for the treatment of DMARD-naive patients with RA. Upadacitinib 15 mg had the highest probability of achieving remission and LDA, followed by tofacitinib 5 mg, baricitinib 4 mg, filgotinib 200 mg, and MTX.

Since they were initially made accessible, the use of JAK inhibitors in the treatment of patients with RA has steadily increased. JAK inhibitor may be a useful therapeutic alternative for DMARD-naive patients with RA. The findings of this network meta-analysis, which included information from direct and indirect comparisons to assess the relative effectiveness and safety of JAK inhibitors, were consistent with the findings of direct comparisons [12, 32, 33, 34]. However, this meta-analysis differed from a previous network meta-analysis conducted by Lee et al. [35] regarding the efficacy and safety of tofacitinib and baricitinib in patients with RA; the previous study included only a specific group of patients with RA who were naive to the JAK inhibitors upadacitinib and filgotinib in addition to DMARD and evaluated the remission and LDA rates. MTX in combination with other biological therapies has been proven to be more successful than MTX alone; nevertheless, other biological monotherapy is no more efficacious than MTX alone against RA [36]. However, in DMARD-naive individuals, monotherapy with JAK inhibitor resulted in a higher remission rate than that with MTX alone. These findings might be significant for individuals who have contraindications to or intolerance to MTX therapy. MTX regimen should be initiated based on the ACR and EULAR recommendations for the treatment of RA [4, 5]. JAK monotherapy, on the other hand, may be an option for patients with RA who have not previously received MTX. It would be of particular interest to include a non-JAK inhibitor in the analysis, e.g., tocilizumab which is also prescribed as monotherapy in RA. Thirty-nine percentage of tocilizumab patients achieved DAS28 remission at 24 weeks compared with 15% of MTX patients [37]. However, we did not include this tocilizumab study because this study did not meet the inclusion criteria.

This network meta-analysis differs from a previous one done by Sung et al. [38] on the relative effectiveness and safety of JAK inhibitors in DMARD-naive RA patients because the endpoints of our research were remission and LDA, while the outcomes of the previous study were the ACR 20% (ACR20), 50, and 70. Our study has certain limitations that may have affected our findings. First, the study analyzed the results of only four RCTs. This is a major concern because the study might have lacked statistical power due to inadequate sample size. Second, 24-week follow-up examination of JAK inhibitors with respect to the remission and LDA rates is inadequate to determine all significant efficacy aspects of JAK inhibitors. Future studies are required with long-term comparative investigation. Third, although the demographics of the patients included in the studies were generally comparable, there was variation in the study design of the included trials. There are differences in mean duration, RF or ACPA positive, and oral steroid usage. Nonetheless, we conducted this network meta-analysis in the context of JAK inhibitors in patients with DMARD-naive RA. As a sensitivity analysis, we performed a random-effect network meta-analysis, which accounts for between-study heterogeneity, since there may be a risk of concluding the ranking of JAK inhibitors. Nonetheless, there are some advantages to this meta-analysis. First, all RCTs included in our network meta-analysis were of excellent quality and significantly consistent. Second, in contrast to the number of patients in different studies being 369–631, our study comprised 2,185 individuals in total. In contrast to individual trials [39, 40], more precise results were obtained due to the increased statistical power and resolution by including separate analyses and predicting the remission and LDA rates of JAK inhibitors in individuals with active RA [41]. This is the first network meta-analysis, to the best of our knowledge, predicting the relative remission and LDA rates of JAK inhibitors in DMARD-naive patients with RA.

In conclusion, we conducted Bayesian network meta-analysis involving four RCTs and determined that upadacitinib is most effective intervention for DMARD-naive patients with active RA and that there was a possible difference in remission and LDA rates among different JAK inhibitors in patients with RA. Long-term trials are required to assess the relative remission and LDA rates of these JAK inhibitors in a larger patient population of DMARD-naive RA.

## Statement of Ethics

An ethics statement is not applicable because this study is based exclusively on published literature.

## Conflict of Interest Statement

The authors have no conflicts of interest to declare.

## Funding Sources

There were no funding sources.

## Author Contribution

Young Ho Lee was involved in conception and design of study, acquisition of data, analysis and/or interpretation of data, drafting the manuscript, and revising the manuscript critically for important intellectual content. Gwan Gyu Song was involved in conception and acquisition of data, analysis and/or interpretation of data, and revising the manuscript critically for important intellectual content.

## Data Availability Statement

All data generated or analyzed during this study are included in this article. Further inquiries can be directed to the corresponding author.

## Figures and Tables

**a:** JAK inhibitors

Study	Subjects	*n* Drugs, mean dosage	Patients, *n*	Achieving remission,^a^ %	Achieving LDA,^b^ %	Mean change of mTSS from baseline
Lee, 2014 [12]	csDMARD-naive	559 Tofacitinib 5 mg, twice daily	373	14.6	27.9	0.2
		MTX 18.5 mg, once weekly	186	7.6	14.0	0.8
Fleischmann, 2015 [13]	csDMARD-naive	369 Baricitinib 4 mg, once daily	159	21^c^	36	0.80
		MTX 17.7 mg, once weekly	210	12^c^	23	1.02
van Vollenhoven, 2018 [14]	csDMARD-naive	631 Upadacitinib 15 mg, once daily	317	48.3	59.9	0.14
		MTX, once weekly	314	18.5	32.2	0.67
Westhovens, 2019 [15]	csDMARD-naive	626 Filgotinib 200 mg, once daily	210	42.4	60.0	−0.04
		MTX up to 20 mg, once weekly	416	29.1	46.2	0.52

**b:** Interventions

Comparison	Study, *n*	Patients, *n*				
MTX	4	1,126				
Tofacitinib 5 mg	1	373				
Baricitinib 4 mg	1	159				
Upadacitinib 15 mg	1	317				
Filgotinib 200 mg	1	210				

MTX, methotrexate; csDMARD, conventional synthetic disease-modifying antirheumatic disease; mTSS, modified total Sharp score.

a DAS28-CRP < 2.6: Disease Activity Score in 28 joints using the C-reactive protein level.

b DAS28-CRP ≤ 3.2.

c DAS28-ESR < 2.6: DAS28 using the erythrocyte sedimentation rate.

**Table 2:** Network meta-analysis of the efficacy of all comparators along with ORs and 95% CrI (range)

Remission: DAS28-CRP <2.6. OR >1 signifies that the treatment in the top left is better				
upadacitinib 15 mg	tofacitinib 5 mg	baricitinib 4 mg	filgotinib 200 mg	MTX
1.95 (0.91–3.94)				
2.12 (1.07–4.21)	1.09 (0.47–2.63)			
2.30 (1.39–3.85)	1.19 (0.59–2.50)	1.09 (0.56–2.14)		
4.13 (2.88–6.02)	2.12 (1.17–4.13)	1.95 (1.10–3.50)	1.79 (1.27–2.53)	

LDA: DAS28–CRP ≤3.2				
upadacitinib 15 mg	tofacitinib 5 mg	baricitinib 4 mg	filgotinib 200 mg	MTX
1.32 (0.72–2.33)				
1.67 (0.95–2.95)	1.27 (0.66–2.49)			
1.81 (1.13–2.90)	1.37 (0.77–2.51)	1.08 (0.61–1.90)		
3.17 (2.29–4.42)	2.40 (1.51–3.98)	1.89 (1.20–3.00)	1.76 (1.25–2.46)	

**Table 3:** Rank probability of tofacitinib, baricitinib, upadacitinib, filgotinib, and placebo efficacy based on the number of patients who achieved a DAS28-CRP <2.6 and DAS28-CRP ≤3.2 response

Treatment	SUCRA
DAS28-CRP <2.6	
Upadacitinib 15 mg	0.985
Tofacitinib 5 mg	0.574
Baricitinib 4 mg	0.506
Filgotinib 200 mg	0.431
MTX	0.004
DAS28-CRP <3.2	
Upadacitinib 15 mg	0.945
Tofacitinib 5 mg	0.699
Baricitinib 4 mg	0.469
Filgotinib 200 mg	0.387
MTX	0.001

SUCRA, surface under the cumulative ranking curve.
